# Effects of xylazine and adrenaline combinations: Preliminary clinical application for non-surgical protocols of nephrosplenic entrapment in horses

**DOI:** 10.14202/vetworld.2021.3188-3193

**Published:** 2021-12-28

**Authors:** Worakij Cherdchutham, Patskit Sukhong, Kanchanog Sae-oueng, Nithisphat Supanwinijkul, Kittanai Wiangnak, Jirayut Srimuang, Tawanhathai Apichaimongkonkun, Sarocha Limratchapong, Soontaree Petchdee

**Affiliations:** 1 Department of Large Animal and Wildlife Clinical Sciences, Faculty of Veterinary Medicine, Kasetsart University, Kamphaeng Saen Campus 73140, Thailand; 2 Kasetsart University Veterinary Teaching Hospital, Faculty of Veterinary Medicine, Kasetsart University, Kamphaeng Saen Campus, 73140, Thailand

**Keywords:** adrenaline, autonomic nervous activity, horse, spleen contraction

## Abstract

**Background and Aim:**

The medical treatment of horses with nephrosplenic entrapment (NSE) of the large colon through administrating phenylephrine and rolling during general anesthesia was effective and less expensive than surgical treatment. However, the selection of drugs for non-surgical treatment of NSE is not a usual method for clinical practice. This study aimed to identify the effects of combined drugs on the cardiac and splenic response in horses and provide information on the NSE of the large colon for clinical application.

**Materials and Methods:**

Six healthy Thai native crossbred horses were enrolled in this study. Horses received two protocols with a withdrawal period of 14 days: Group 1 received xylazine (0.5 mg/kg IV) and adrenaline (1 mcg/kg IV), and Group 2 received xylazine (0.5 mg/kg IV) and adrenaline (3 mcg/kg IV). Heart rate (HR), HR variability (HRV), heart dimensions, and the splenic response of six horses were measured before the sedation, 30 and 60 min later, and 65, 70, 75, 80, 90, and 100 min after adrenaline administration. Doppler was used to obtain systolic blood pressure.

**Results:**

The HRV low-frequency and high-frequency power ratios decreased after using xylazine. Hypertension was observed after adrenaline administration. In this study, there were only minimal differences in the HR and respiratory rate between groups. However, overall cardiac and splenic parameters were statistically higher in Group 2.

**Conclusion:**

This study suggested that xylazine and three micrograms of adrenaline preserved the cardiac autonomic activity balance and were safe to use non-surgical applicability in horses.

## Introduction

There are several protocols for treating nephrosplenic entrapment (NSE) of the large colon in horses. The surgical procedure has the disadvantages of high cost, high morbidity, and long-term recovery [1,2]. In the actual situation, a non-surgical approach of the patient with NSE includes an intravenous administration of fluid supportive, analgesic agents, exercise, and rolling under general anesthesia [3-5]. In addition, a non-surgical treatment by administering alpha-adrenergic agents is a treatment procedure for NSE of the large colon in horses. Alpha-adrenergic agents such as phenylephrine were used to promote splenic contraction in the non-surgical treatment of NSE in horses. After alpha-adrenergic agents administration, the splenic contraction resulted in an enlargement of the space between the spleen and the abdominal wall, thus increasing the possibility of the left colon moving back into its natural position. Alpha-2 adrenergic agonists such as xylazine and detomidine are short-acting sedatives commonly used for analgesic properties in horses [6-8]. The disadvantages of xylazine include ataxia, bradycardia, arrhythmias, and cardiac output reduction [9]. The previous study reported that alpha-2 adrenergic receptor agonists combined with epinephrine did not interfere with the duration of epinephrine-induced spleen contraction in horses with NSE [10]. The blood lactate measurement has been used to evaluate prognosis in equine and human medicine. Blood lactate concentrations were used as an indicator of tissue perfusion, oxygen delivery, and the presence of a severe inflammatory response. Therefore, the blood lactate measurement could be a valuable therapeutic guide and prognosis of horses receiving surgical treatment.

Heart rate variability (HRV) has been suggested to measure a non-invasive index for autonomic nervous control. A previous study showed that cardiac condition progression could alter cardiac autonomic activity, leading to increased HR and decreased HRV [11-13]. In addition, the previous study reported that HRV was associated with ischemic gastrointestinal disease and non-survival after the treatment. HRV analysis provides diagnostic and prognostic information to manage the horse with severe gastrointestinal disease [14]. Many studies used various anesthetic agents, but no studies have been performed to analyze the cardiac autonomic activity using HRV in response to desire the agents with minimal cardiovascular effects in horses [15].

This study aimed to evaluate the effects of xylazine and adrenaline on cardiac autonomic nervous activity using HRV analysis in horses and provide information on the non-surgical NSE protocols of the large colon for clinical application. This study hypothesized that (i) the effects on the cardiovascular system, respiratory rate (RR), and splenic responses of xylazine and adrenaline could be characterized using HRV, echocardiography, and abdominal ultrasonography. (ii) Adrenaline produces a dose related to the cardiovascular and splenic response.

## Materials and Methods

### Ethical approval

This study was approved by the Ethical Committee for Animal Experiments, Faculty of Veterinary Medicine, Kasetsart University, approval number (ACKU62-VET-061).

### Study period and location

This study was conducted from January 2018 to April 2019 at Kasetsart University Veterinary Teaching Hospital.

### Animals

**Figure-1:**
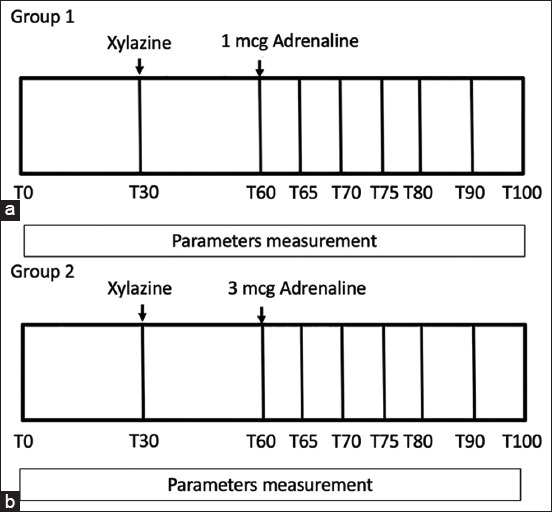
(a and b) In the study protocol, all parameters were evaluated before administering xylazine (T0) and immediately after xylazine administration (T30), immediately after adrenaline administration (T60), and at 5 and 10 min intervals until 100 min.

Six Thai native crossbred gelding horses aged 9.8±1.1 years, weighing 266.3±9.2 kg, were used for this study. All horses were healthy based on the clinical examinations, echocardiography, and the routine blood test, the horse with cardiovascular disease, were excluded from the study. We designed a cross-over study, and horses were assigned in a randomized order into two protocols with a withdrawal period of at least 14 days between two procedures. Horses in Group 1 were administered with xylazine (0.5 mg/kg IV) and adrenaline (1 mcg/kg IV). Horses in Group 2 underwent the protocol using xylazine (0.5 mg/kg IV) and adrenaline (3 mcg/kg IV). Xylazine and adrenaline were given 30 min apart in both protocols. All horses were then maintained for approximately 100 min with a continuous recording of all parameters for further analysis using a blinded assessment, as illustrated in Figure-1.

### Cardiovascular variables

The echocardiographic examination (Mindray, China) with 2 MHz transducers was done to rule out cardiac pathology and evaluate heart contractility functions. The left ventricular wall structure and function were calculated by measuring the images from a two-dimensional plane as in the previous study [15]. Cardiac parameters such as HR, stroke volume, cardiac output, fractional shortening, and left ventricle internal diameter were evaluated offline.

Non-invasive cardiac autonomic nervous control measurements were performed on six horses using a Holter electrocardiography (ECG) recording device (BTL Medical Technologies, Thailand). Five electrodes were placed on the thorax’s shaved skin with elastic tape, as previously described, to provide the 100 min ECG recording for the baseline and after administered xylazine and adrenaline [15]. The HRV parameters were recorded and analyzed before drug administration (baseline), 30 min (T30), and 100 min (T100) after drug administration with an analyzing program (BTL Medical Technologies). HRV contains time and frequency domains. The time-domain parameters are measured from the peak of the N-N intervals, or the ECG’s R-R peak, which can be calculated as the standard deviation of the N-N intervals (SDNN) and the root mean square of successive differences in R-R intervals (RMSSD). The frequency-domain or spectral analysis is measured from the transformed ECG as a spectral signal. The frequency-domain parameters were expressed as the high frequency (HF) (0.15-0.5 Hz) and low frequency (LF) (0.04-0.15 Hz).

### Spleen dimension measurements

The dorsocaudal to ventral-cranial edges (L1) and dorsocaudal to ventrocaudal edges (L2) of the spleen were localized on the abdominal surface using a 2.0 MHz transducer (Mindray). The distance between the marks on the skin was measured over time using measuring tape described in a previous study [10].

### Packed cell volume (PCV) measurements

The blood samples were collected from all horses from the jugular vein, and plasma lactate was determined immediately using a blood lactate analyzer (blood lactate test meter, XPER Technology, Taiwan). After that, the blood samples were placed into bottles containing an anticoagulant (ethylenediaminetetraacetic acid). PCV was measured using a capillary tube and centrifuged at 10,000 revolutions per minute for 5 min. Non-invasive blood pressure was measured with a Doppler using the cuff positioned at the middle coccygeal cavity as previously described [16].

### Statistical analysis

Normal distribution was calculated using a histogram of the data. All data are shown as mean±standard error of the mean. Statistical analysis of a cross-over design was performed using a repeated one-way and two-way analysis of variance (ANOVA) (GraphPad Prism software version 5). p=0.05 or less was indicated for statistical significance.

## Results

**Figure-2:**
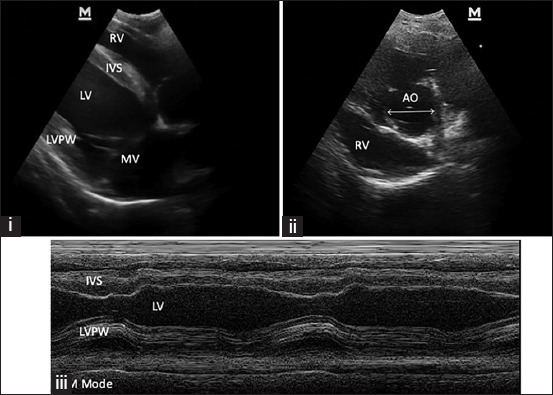
Echocardiographic of a longitudinal image of the heart (i), (ii) and motion mode (M mode) image of the left ventricle (iii); RV=Right ventricle, LV=Left ventricle, IVS=Interventricular septum, LVPW=Left ventricular proximal wall, MV=Mitral valve, and AO=Aorta.

**Figure-3:**
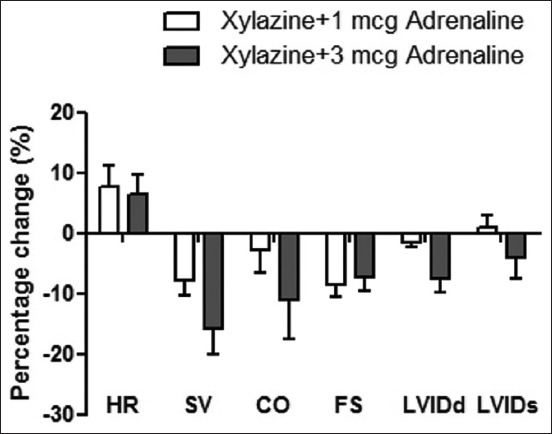
The comparisons of percentage change of cardiac parameters at baseline (T0) and at T100; HR=Heart rate, SV=Stroke volume, CO=Cardiac output, FS=Fractional shortening, LVIDd=Left ventricle diameter during diastole, and LVIDs=Left ventricle diameter during systole; error bars indicate standard error of the mean.

All enrolled six horses (aged 9.8±1.1 years, weighing 266.3±9.2 kg) remained in the study without significant complications. The effects of adrenaline administration were evaluated at various times within each group, such as sweating, tachycardia, and restlessness. The results of HRV are summarized in Table-1. Blood lactate levels increased after combined drugs were administered in both groups when compared to the baseline; Group 1 from 1.13±0.12 to 1.68±0.17 and Group 2 from 1.58±0.3 to 2.16±0.19, horses in Group 2 had a higher lactate level than horses in Group 1. The cardiac output in both groups was within the normal limit. Only minimal changes in the echocardiography parameters were observed in the present study (Figure-2), and there were no significant differences between the groups. However, horses in Group 1 had a higher percentage change in HR values (7.64% vs. 6.4%; Figure-3).

**Table 1:** Heart rate variability at T0, T30, and T100.

Time domain	T0	T30	T100
RR (ms)			
Group 1	1425.33±354.43	2143.67±507.48	367.17±147.89
Group 2	1119.17±358.53	2375.67±501.58	2393.17±478.86
SDNN (ms)			
Group 1	147.67±60.81	137.0±55.27	186.33±70.78
Group 2	145.67±56.08	94.67±24.63	125.50±20.21
RMSSD (ms)			
Group 1	940.33±76.66	873.67±91.35	817.33±87.31
Group 2	924.67±172.66	923.67±117.25	1009.33±113.53

**Frequency domain**	**T0**	**T30**	**T100**

LF			
Group 1	1.66±0.25	2.18±0.31	2.10±0.52
Group 2	1.32±0.18	1.99±0.87	1.98±0.42
HF			
Group 1	2.10±0.55	2.89±0.58	1.81±0.54
Group 2	1.39±0.16	2.87±0.89	1.85±0.39
LF/HF ratio			
Group 1	0.80±0.13	0.79±0.08	1.13±0.16
Group 2	0.91±0.15	0.66±0.05	1.01±0.09

RR=The ECG’s R-R peak; SDNN=Standard deviation of the R-R intervals; RMSSD=The root mean square of successive differences in R-R intervals. The frequency domain of the spectral analysis is represented as the ratio of LF and HF domain. Two-way ANOVA revealed no statistically significant interaction between the dose of adrenaline and time on the HRV. Data are shown as mean±SEM. LF=Low frequency, HF=High frequency

**Figure-4:**
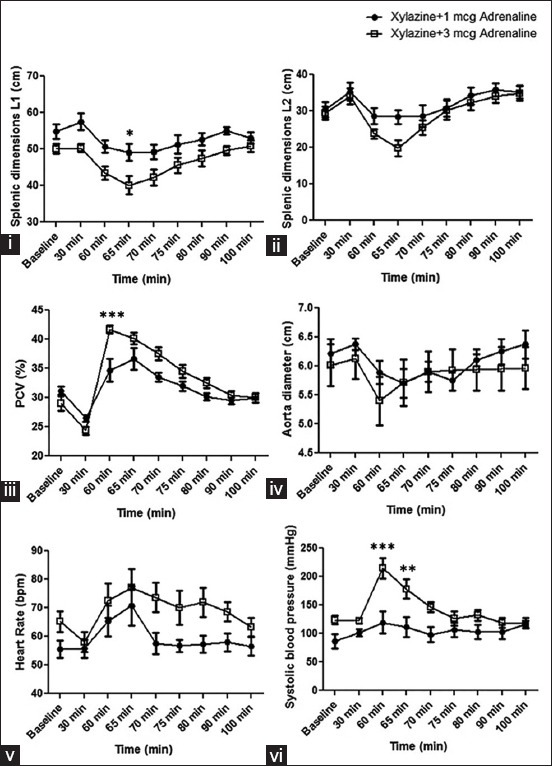
Two-way analysis of variance revealed an interaction of xylazine (0.5 mg/kg IV) and differences dose of adrenaline (1 mcg and 3 mcg IV) on (i) splenic dimensions; L1=Dorsocaudal to ventrocranial splenic edges; (ii) L2=Dorsocaudal to ventrocaudal splenic edges; (iii) pack cell volume; (iv) aorta diameter; (v) heart rate; (vi) systolic blood pressure; before drug administration (baseline), 30 min (T30), 60 min (T60), 65 min (T65), 70 min (T70), 75 min (T75), 80 min (T80), 90 min (T90), and 100 min (T100) after drug administration, *p<0.05, **p<0.01, ***p<0.001 versus the baseline.

All horses showed a marked decrease in the splenic length 5 min after administration of adrenaline. Two-way ANOVA revealed a significant interaction between the dose of adrenaline, and statistically significant differences (*p<0.05) were found, as shown in Figure-4 (i). After adrenaline administration, splenic length (L1 and L2) markedly decreased from a baseline value of 50.08±1.25 cm to 40.08±2.50 cm, and 29.42±1.72 cm to 19.83±2.18 cm in Group 2, respectively. The decrease in splenic dimensions was accompanied by a decrease in aortic diameter in both groups. PCV increased in Group 2 compared with baseline after adrenaline administration. There are statistical differences in the PCV concentration compared with the baseline and compare between groups after the drug administration (***p<0.001, Figure-4 (iii)). Results revealed no significant difference between the dose of adrenaline on the RR, RR decreased but remained within normal physiological limits after xylazine administration, and no dyspnea was observed before and after the combined drug administration, which is similar to the previous study [17].

HR and systolic blood pressure (SBP) increased within 1 min after adrenaline were administered in both groups. Two-way ANOVA revealed a significant interaction between the dose of adrenaline (****p<0.0001). SBP was increased in both protocols and statistically significant differences (***p<0.001 and **p<0.01 were found at 60 and 65 min after adrenaline administration Figure-4 [vi]). The result from this study showed that adrenaline produces a dose-dependent effect on the cardiovascular and splenic response.

## Discussion

This present study showed that a protocol using xylazine (0.5 mg/kg IV) + adrenaline (3 mcg/kg IV) could apply in the horse, producing higher spleen contraction ability but less adverse effect on heart contraction and cardiac dimension than using xylazine (0.5 mg/kg IV) + adrenaline (1 mcg/kg IV). Although there have been previous trials in using phenylephrine and xylazine for non-surgical treatment of NSE in horses, adrenaline and xylazine were used in this study because it is inexpensive and widely used in veterinary clinical practice. However, phenylephrine is typically used in human medicine and is commonly used in veterinary medicine. Our study suggested that adrenaline could be an alternative safety drug that can be used instead of phenylephrine, which may be difficult to find in some countries such as Thailand. HR and SBP increased after adrenaline was administered in both groups. Horses in Group 1 had a higher change in HR values. PCV significantly decreased after xylazine administration and increased after adrenaline administration, similar to the present study [18].

Blood lactate concentrations are often monitored in sick horses. A previous study suggested that lactate concentrations measurement is a practical prognostic guide and valuable for therapeutic interventions, particularly early fluid therapy [19]. The previous study reported that the plasma lactate concentration increased during general anesthesia with alpha-2 agonist agents [20]. Therefore, it is essential to know whether the administration of adrenaline and xylazine impacts blood lactate concentrations in horses. In this study, blood lactate levels increased after adrenaline were administered in both groups. Horses in Group 2 had a higher lactate level than horses in Group 1. However, the pathophysiology of the increase in the blood lactate concentration is likely multifactorial [14], although alpha agonist-induced hyperglycemia and hyperinsulinemia may likely play roles. Therefore, monitoring the blood lactate concentrations should be performed when making decisions regarding the diagnosis, care, and prognosis of horses receiving alpha-agonist agents.

A cardiovascular assessment such as HRV, blood pressure, and contraction function will help identify significant factors influencing cardiovascular health during anesthesia and recovery. Therefore, a cardiovascular examination should be a part of every anesthetic protocol. Moreover, the contraindications in non-surgical treatment using xylazine and adrenaline combinations are that the treatment should not be used in aged horses. Furthermore, the horse should not have any cardiovascular disease such as myocardial infarction or aortic valve aneurysm [21,22]. In addition, it should be aware of an associated risk of hemorrhage. Then, it is recommended to have a cardiovascular monitoring and echocardiography test before using phenylephrine and xylazine for non-surgical treatment of NSE in horses [23]. In the present study, only one cardiologist has done echocardiography before the study to test that the horses had no cardiovascular disease.

The SDNN decreased after xylazine administration in both groups (Table-1), indicating a reduction in parasympathetic tone related to the slight increase in HR. RMSSD increased in Group 2, which reflected the faster cardiac recovery than Group 1. A decrease in LF power in both groups after adrenaline administration indicated vagal inhibition. This study showed that combined drugs in both protocols increased HR and affected parasympathetic activity as a previous study [15]. The higher decrease in HF power in Group 1 indicated a high parasympathetic activity, and horses in this group had high LF/HF ratio values compared with the other group. The results suggested that xylazine (0.5 mg/kg IV) + adrenaline (3 mcg/kg IV) on Group 2 might better preserve cardiac autonomic activity balance than 1 mcg of adrenaline (Group 1). A previous study showed that the reduced HRV is associated with ischemic gastrointestinal disease and non-survival. However, HRV appeared to be influenced by age and environmental factors such as hot and humid environments [24]. HRV may provide diagnostic and prognostic information in horses with gastrointestinal diseases [14]. In addition, HRV can be used to select an endurance horse with the fastest cardiac recovery. The results of our present study suggested that HRV may use as a parameter for anesthetic risk assessment for non-surgical treatment of NSE in horses. However, there was a variation in the time domain, but no significant HRV results in the frequency domain were observed. The results showed that the ECG Holter to test the frequency domain of HRV in horses was not considerably affected by the two protocols. The overall cardiac functions were more highly decreased in Group 2, and there was a slight difference between the cardiac dimensions of the two protocols. However, there was no significant difference between Groups 1 and 2 related to cardiac functions and heart dimensions (Figure-3). This study showed that the horses were significantly influenced concerning the aortic diameter and splenic dimension (Figure-4). Similar to our study, a previous study suggested that the spleen dimensions (L1 and L2) were assessed using two long axes with the same dorsocaudal edge. This protocol allowed repeated measures at short time intervals with a minimal number of marks; the study suggested no need to consider either the spleen surface, spleen length in a single dorsoventral axis, and the spleen volume [10].

In our pilot study, an insufficient number of horses in each group could affect the statistical analysis, which was a limitation. Although the animal’s number should increase for analyzing our data, we still gain valuable results in understanding the cardiovascular response induced by the combined drug treatment. Another limitation in this study did not have real clinical cases. The clinical research established by the actual case can mimic the pathophysiological changes during the NSE situation. However, the actual situation model has a disadvantage in the number of animals than the study on a laboratory animal.

This study suggested that xylazine and three micrograms of adrenaline preserved the cardiac autonomic activity balance and can be used for non-surgical applicability in horses. Based on the results in our study, it is recommended to use xylazine and three micrograms of adrenaline for non-surgical treatment of NSE in horses. However, this study was performed on a healthy horse, and further investigations are required to ensure validity for future applications.

## Conclusion

The study showed that xylazine (0.5 mg/kg IV) + adrenaline (3 mcg/kg IV) could be used in the horse, producing higher spleen contraction but less adverse effect on heart contraction and cardiac dimension than another protocol. The results suggested that the effects on the cardiovascular, respiratory, and splenic responses of xylazine and adrenaline could be characterized using HRV, echocardiography, and abdominal ultrasonography. In addition, the result from this study showed that adrenaline produces a dose related to the cardiovascular and splenic response.

## Authors’ Contributions

WC and SP: Contributed to the conception and design of the study. SP: Wrote the manuscript. WC and SP: Contributed to data collection. WC, PS, KS, NS, KW, JS, TA, SL, and SP: Participated in laboratory analysis of the samples. SP: Performed analytical work. SP: Critically revised the manuscript. All authors read and approved the final manuscript.
